# Audit of the Use of Outcome Measures Within Child and Adolescent Mental Health Outpatient Services in Rotherham, Doncaster and South Humber NHS Foundation Trust

**DOI:** 10.1192/bjo.2024.544

**Published:** 2024-08-01

**Authors:** Lydia Bell, Mohan Thomas

**Affiliations:** Rotherham, Doncaster and South Humber NHS Foutdation Trust, Rotherham, United Kingdom

## Abstract

**Aims:**

Rotherham, Doncaster and South Humber NHS Foundation Trust (RDaSH) has 28 Promises as part of its Strategy.

Promise 16 is to: *Focus on collating, assessing and comparing the outcomes that our services deliver, which matter to local people, and investing in improving those outcomes year on year.*

This audit in November 2023 looked at the practice of using outcome measures for CAMHS patients in order to highlight areas of development for the service to work toward achieving the promise.

**Methods:**

We wanted to understand if young people were having outcome measures completed and if so, when, what and how often. We achieved this by using a dip sample of five patients each across the three different localities (Rotherham, Doncaster and Scunthorpe).

A report was generated to include all patients discharged from CAMHS in the preceding three months to September 2023. Young people who had been with the service less than six months were excluded from the audit. Five patients were chosen randomly from each locality and their electronic patient record on System One was studied.

Information in the patient records was compared against the audit standards and recorded in Excel so the data could be analysed.

**Results:**

The results showed that 11 of 15 young people had an outcome measure completed at some point during their episode of care. All five young people in Scunthorpe had an outcome measure recorded in their clinical records however this tended to only happen at the very start, meaning there was no basis for comparison. Four out of five patients in Doncaster had outcome measures in the clinical record and these were undertaken throughout the episodes of care. In Rotherham, two of five young people had outcome measures recorded in the clinical records.

The most frequently used outcome measure was the RCADS but the SDQ was also used.

**Conclusion:**

There is work to be done to ensure the use of outcome measures becomes routine, and also to standardise both the type and frequency of use. The Trust is aiming to increase their use by utilising SystmOne's capabilities to interface with service user mobile devices to send out outcome measures to patients. There is also a plan to inform staff within the service about the expected use of outcome measures. This audit will be repeated in 2024 to see if the Trust are moving closer to delivering their promise.

